# The Psychological Impact of COVID-19 Pandemic on People With Multiple Sclerosis

**DOI:** 10.3389/fneur.2020.580507

**Published:** 2020-10-30

**Authors:** Francesco Motolese, Mariagrazia Rossi, Giuliano Albergo, Domenica Stelitano, Marialucia Villanova, Vincenzo Di Lazzaro, Fioravante Capone

**Affiliations:** ^1^Unit of Neurology, Neurophysiology, Neurobiology, Department of Medicine, Università Campus Bio-Medico di Roma, Rome, Italy; ^2^NeXT: Neurophysiology and Neuroengineering of Human-Technology Interaction Research Unit, Campus Bio-Medico University, Rome, Italy

**Keywords:** multiple sclerosis, COVID - 19, anxiety, depression, sleep quality, fatigue, social isolation

## Abstract

**Objective:** The coronavirus disease 2019 (COVID-19) has radically changed the world in a few weeks. Italy has been one of the first and most affected countries with more than 30,000 deaths up to now. Public health measures as quarantine or national lockdown are necessary to limit the spread of infectious diseases, but it is unsurprising that depriving people of their liberty has negative psychological effects. This is especially the case for people with chronic diseases, including neurological conditions like multiple sclerosis (MS). People with MS (PwMS) have a higher burden of neuropsychiatric comorbidities and are known to undertake maladaptive coping strategies in stress conditions. The aim of the present study is to investigate the impact of COVID-19 pandemic lockdown on mental health of an Italian cohort of PwMS in comparison with healthy controls (HCs).

**Methods:** A total of 60 PwMS and 50 HCs (chosen among patients' cohabitants) were asked to answer a Web-based survey. This survey inquired about the impact of COVID-19 on patient's quality of life, job, and daily routine. Mood, fatigue, and sleep quality were evaluated using the Beck Depression Inventory II (BDI-II), the Generalized Anxiety Disease 7 (GAD-7), the Fatigue Severity Scale (FSS), and the Pittsburgh Sleep Quality Index (PSQI).

**Results:** Overall, patients had higher scores of BDI, FSS, and PSQI, and these differences were statistically significant (*p* < 0.05). When we looked at the subscores of the BDI, we detected a statistically significant difference for the neurovegetative part—that concerns with sleep, appetite, sex, and quality of sleep (*p* < 0.05). One out of five patients reported new symptoms or worsening of known symptom, in particular, sensory disturbances, and fatigue. However, no symptoms were severe enough to require hospitalization. When we looked for correlations among variables, we found that there was a significant relationship between unemployment and BDI total score, GAD-7, and PSQI in MS group. The presence of new symptoms or the worsening of symptoms positively related to FSS and to PSQI.

**Discussion:** We identified that the severe acute respiratory syndrome coronavirus 2 (SARS-CoV-2) pandemic had a significant impact on the psychological status of patients with MS. Compared with the general population, PwMS presented a higher burden of depressive symptoms, a worse sleep quality and perceived an increase in fatigue level, one of the most disabling symptoms of MS. The COVID-19 epidemic poses a challenge to psychological resilience. More studies are warranted to better understand the long-term consequences of the pandemic on mental health of vulnerable people during the disease outbreaks.

## Introduction

The coronavirus disease 2019 (COVID-19)—the disease caused by severe acute respiratory syndrome coronavirus 2 (SARS-CoV-2), a novel strain of coronavirus—has radically changed the world in a few weeks. Initially, the perception was that the epidemic would be localized in China only, but then the virus has spread with alarming speed and a pandemic quickly developed. Italy has been one of the first and most affected countries with more than thirty thousand deaths up to now. Accordingly, on March 2020, for about 3 months, the Italian government imposed a national lockdown, thus limiting free movement of people and shutting down all the activities considered not essential. Public health measures as quarantine or national lockdown are necessary to limit the spread of infectious diseases, but it is unsurprising that depriving people of their liberty has negative psychological effects (1). Besides, many people were forced to self-isolate and some experienced financial difficulties due to economic crisis. COVID-19 has then deeply affected human communities around the world, and there is fear that people with chronic diseases are more vulnerable to negative psychological effects (2).

Multiple sclerosis (MS) is a chronic inflammatory disease of the central nervous system (CNS) that affects more than 2 million people worldwide. The mean age of diagnosis is 30 years and, in most patients, the clinical course is characterized by periodic neurological relapses (i.e., relapsing-remitting type), eventually leading to progressive neurodegeneration after about one or two decades after disease onset (i.e., secondary progressive type) (3–5). Apart from the most known clinical manifestations, such as diplopia, optic neuritis, sensory loss, or limb weakness, cognitive, and neuropsychiatric dysfunctions in people with MS (PwMS) can frequently occur (6). In particular, depression, and anxiety are frequently reported by people affected by chronic diseases, but they occur with higher rates in PwMS, with a dramatic impact on quality of life and often influencing compliance to treatments (7–10). Neuropsychiatric comorbidities often present in association with fatigue, one of the most disabling symptoms of MS, experienced by almost 80% of patients during the entire course of the disease (11).

Brain network alterations, inflammatory changes, intrinsic predisposing factors, and psychosocial elements are the main pathophysiological determinants for the development of fatigue and mood disorders in MS (8, 12). So, it is not surprising that PwMS are more vulnerable to psychosocial stressors and, although results from different studies are controverted and no definitive conclusions can be drawn, stress is considered a potential predictor of MS relapse and onset (13). Indeed, chronic stress influences immune functioning that in turn alters dopamine release in the mesolimbic pathway, a key neural network involved in reward interest (14). Accordingly, the ability to cope with stressors is dramatically impaired in PwMS. The COVID-19 pandemic can be seen as a testing bench for mood disorders in PwMS.

The aim of the present study is to evaluate the impact of COVID-19 pandemic lockdown on the neuropsychiatric profile of an Italian cohort of PwMS in comparison with HCs.

## Methods

A total of 75 healthy controls (HCs) and 75 patients affected by MS were randomly selected from the outpatient clinic of Policlinico Universitario Campus Bio-Medico di Roma to participate in an online survey. The sample size was calculated on the basis of the estimated prevalence of neuropsychiatric symptoms in PwMS and HCs (6, 15). To detect a difference in incidence of neuropsychiatric symptoms of at least 30% in the two groups with a significance level of 5% and a power of 80%, 104 participants in total were required. All patients had a previous visit to our outpatient clinic not older than 6 months. HCs were chosen among patients' cohabitants or were family members or partners. The main demographical features of the two groups are summarized in Table 1.

**Table 1:** Main demographic and clinical features of participants.

	**Patients** **(*n* = 60)**	**Healthy controls** **(*n* = 50)**	***p* value**
**Age**			
<50 years [*n* (%)]	40 (66.7)	34 (68)	NS
> 50 years [*n* (%)]	20 (33.3)	16 (32)	NS
Sex [F; *n* (%)]	41 (68.3)	31 (62)	NS
**Education**			
Higher education [*n* (%)]	9 (15)	24 (48)	NS
High school [*n* (%)]	32 (53.3)	16 (32)	0.047^*^
Upper secondary school or lower [*n* (%)]	19 (31.7)	10 (20)	NS
**Work status**			
Currently employed [*n* (%)]	35 (58.3)	31 (62)	NS
Unemployed [*n* (%)]	25 (41.7)	19 (38)	NS
**Marital status**			
Married	26 (43.3)	24 (48)	NS
Cohabitation	7 (11.7)	4 (8)	NS
Engaged	12 (20)	12 (24)	NS
Divorced	4 (6.7)	2 (4)	NS
Single/widowed	11 (18.3)	8 (16)	NS
Previous diagnosis of major depressive disorder [*n* (%)]	9 (15)	8 (16)	NS
**Disease type**			
Relapsing remitting [*n* (%)]	47 (78.3)	–	
Primary progressive [*n* (%)]	4 (6.7)	–	
Secondary progressive [*n* (%)]	9 (15)	–	
Disease duration [years, mean (SD)]	5.1 (5.9)	–	
**Disease-modifying drugs (DMDs)**			
Dimethyl fumarate [*n* (%)]	22 (36.7)	–	
Glatiramer acetate [*n* (%)]	7 (11.7)	–	
Teriflunomide [*n* (%)]	8 (13.3)	–	
Interferon-Beta [*n* (%)]	8 (13.3)	–	
Natalizumab [*n* (%)]	5 (8.3)	–	
Anti-CD20 antibodies [*n* (%)]	5 (8.3)	–	
None [*n* (%)]	5 (8.3)	–	
BDI total score [mean, (SD)]	9.7 (8.9)	6.9 (8.1)	0.010^*^
BDI neuroveg subscore [mean, (SD)]	4.5 (3.0)	3.1 (3.4)	0.006^*^
BDI cognitive subscore [mean, (SD)]	5.3 (6.7)	3.7 (5.2)	0.152
FSS mean score [mean, (SD)]	3.6 (1.6)	2.5 (1.4)	0.001^*^
GAD-7 score [mean, (SD)]	13.8 (4.3)	15.2 (4.7)	0.331
PSQI total score [mean, (SD)]	6.9 (3.7)	4.7 (2.7)	0.001^*^

*NS, not significant*.

* *p <0.05*.

All subjects were contacted initially by phone calls, then followed by an e-mail containing the link to the survey and all the necessary instructions if they agreed to participate. The recruitment process took place over a period of time from the end of April to the beginning of May, before the end of the lockdown. All patients enrolled in the study provided informed consent by means of an online form. This study was performed according to the Declaration of Helsinki, and it was approved by the local ethics committee.

Web-based surveys have been increasingly used in the last few years as a tool for data collection. However, such surveys can be subject to considerable bias—for instance, the nonrepresentative nature of the Internet population. The Checklist for Reporting Results of Internet E-Surveys (CHERRIES) is a checklist for ensuring the quality of studies that utilizes Web-based surveys (16). In this study, data collection and report are compliant with the CHERRIES Web-surveys guidelines (Supplementary Table 1).

We designed a survey divided in 12 sections using the online platform provided by Google LLC (Mountain View, California, USA) (i.e., Google Form). Section 1 included the informed consent part. Sections 2 and 3 provide sociodemographic data. Section 4 included questions about mood, previous diagnosis of depression, and the use of psychopharmacological therapies. Sections 5 and 6 talk about the impact of the COVID-19 pandemic lockdown on subject's life. Section 7 specifically focused on patients with MS and included questions about disease subtypes, disease duration, symptoms, and disease-modifying drugs. Section 8 investigated the impact of COVID-19 pandemic on patient's scheduled follow-up visits or infusions. Finally, Sections 9, 10, 11, and 12 included the Beck Depression Inventory II (BDI-II) (17, 18), the seven-item Generalized Anxiety Disease (GAD-7) (19), the Fatigue Severity Scale (FSS) (20), and the Pittsburgh Sleep Quality Index (PSQI) (21), respectively.

The BDI-II is a psychometric test that measures the severity of depressive symptoms. Despite some concerns about the validity of the items exploring “somatic symptoms” (e.g., fatigue) (22), BDI-II is a widely used self-report questionnaire with a sensitivity of 85% and a specificity of 76% for detecting clinically significant depression in PwMS (23).

GAD-7 is a brief questionnaire based on generalized anxiety disorder criteria found in the DSM-IV and its internal validity and reliability (Cronbach alpha = 0.75) has been demonstrated also in PwMS (19). Different tools to evaluate fatigue in MS exist, but FSS is one of the most used questionnaires worldwide. Despite being brief and dealing mainly with the physical aspects of fatigue, it shows good internal consistency (Cronbach alpha = 0.93—possibly indicating some redundancy) and validity, making it a good screening tool for fatigue (24). Finally, the PSQI is a self-report questionnaire that measures sleep quality over a 1-month time interval. A PSQI score <5 is shown to have a diagnostic sensitivity of 89.6% and a specificity of 86.5% to identify the so-called poor sleepers (25). PSQI has been demonstrated to be a reliable and valid tool to evaluate sleep quality also in PwMS (25).

Both patients and HCs received two different links to the same survey, but HCs could automatically skip the part about MS history. To avoid missing data, we made all questions mandatory, a feature included in Google Form; besides, after completing the survey, the link expired, thus preventing duplicate entries. Filling out the survey took 10–15 min. All data were completely anonymized.

The primary endpoint of the study was to detect any difference of BDI total score and BDI subscores, GAD-7, FSS, and PSQI score among the two groups. Secondary endpoints aimed to investigate if demographical and clinical data correlated with BDI total score and BDI subscores, GAD-7, FSS, and PSQI score.

### Statistical Analysis

All statistical analyses were performed using the software SPSS, v 25.0 (IBM Co., Armonk, NY). Data were analyzed for normality of distribution using the Kolmogorov-Smirnov test and were expressed as mean (± standard deviation (SD)) for continuous variables or as frequencies (*n*, %) for categorical variables. The Mann-Whitney rank sum test was used to compare continuous variables between group (patients with MS vs. HCs). Spearman's Rho coefficients were calculated to estimate correlation strengths between variables. All statistical tests were two tailed, and statistical significance was defined as *p* value <0.05.

### Data Availability

Anonymized data will be shared with qualified external researchers, after approval of their requests.

## Results

**Figure 1:**
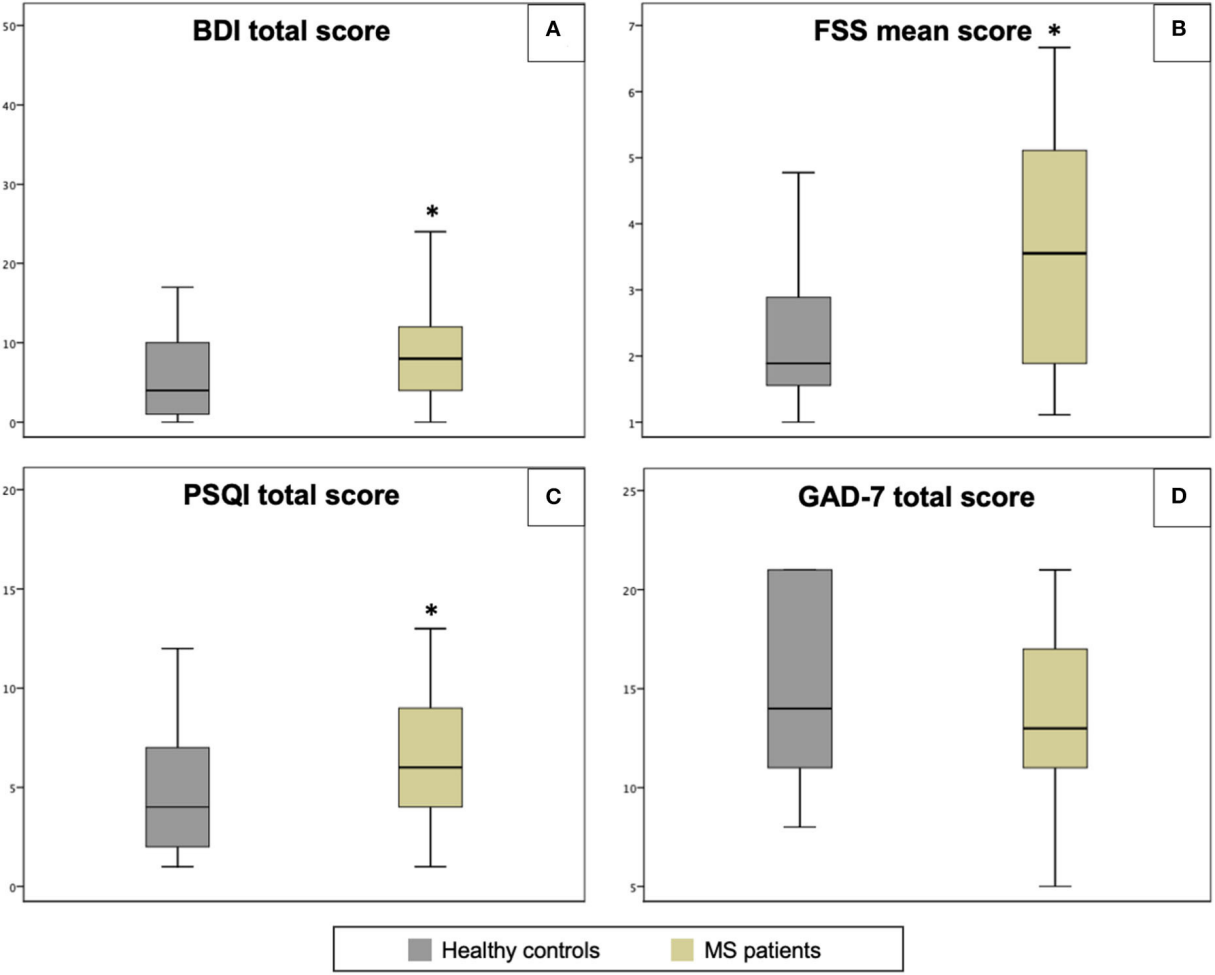
Comparisons of questionnaire scores between patients and healthy controls (HCs). BDI-II, Beck Depression Inventory II **(A)**; FSS, Fatigue Severity Scale **(B)**; PSQI, Pittsburgh Sleep Quality Index **(C)**; GAD-7, the Generalized Anxiety Disease 7 **(D)**. Black line within the box marks the median. *p < 0.05.

Results and demographical data are summarized in Table 1. One hundred and fifty subjects received the link to the survey and one hundred and ten of them actually participated (60 patients and 50 HCs). Overall, patients had higher scores of BDI, FSS, and PSQI, and these differences were statistically significant (Figure 1). When we looked at the subscores of the BDI, we detected a statistically significant difference between patients and HCs (4.5 vs. 3.1, respectively) for the neurovegetative part—that concerns with sleep, appetite, sex, and quality of sleep (*p* < 0.05).

In the MS group, 18 patients out of the 46 (39.1%) who answered the question about the impact of the pandemic on their job, reported that COVID-19 did have a negative impact, especially in terms of working hour reduction (67.4% of them). Similarly, 7 HCs out of 33 (21.2%) reported an impact on their job, also in this case in terms of working hour reduction (46% of HCs). Most subjects worked from home (i.e., smartworking) (56.7% of patients *vs*. 46.7% of HCs), while 43.2% of patients and 53.3% of HCs kept going to their usual workplace. Not surprisingly, the time spent at home increased compared with the prelockdown period of time, with 56.5% of patients with MS and 52% of HCs reporting to spend the entire week at home. No patient or HCs reported COVID-19 symptoms and only 7 out of 110 had an oropharyngeal swab that resulted negative in all cases. Eight patients (13.3%) had a scheduled infusion that was postponed because of COVID-19. Twelve (20%) patients reported new symptoms or worsening of known symptom, in particular, sensory disturbances (50%) and fatigue (33%). However, no symptoms were severe enough to require hospitalization. Thirty-four patients (57%) reported a clinically significant fatigue, as measured by a FSS mean score ≥4.

When we looked for correlations between variables, we found that there was a significant relationship between unemployment and BDI total score (*r*_s_ = 0.552; *p* < 0.05), FSS (*r*_s_ = 0.406; *p* < 0.05), GAD-7 (*r*_s_ = 0.452; *p* < 0.05), and PSQI (*r*_s_ = 0.401; *p* < 0.05) in the MS group. Besides, the use of antidepressive drugs positively related to BDI total score (*r*_s_ = 0.349; *p* < 0.05), FSS (*r*_s_ = 0.276; *p* < 0.005), GAD-7 (*r*_s_ = 0.242; *p* < 0.05), and PSQI (*r*_s_ = 0.334; *p* < 0.005). Surprisingly, a previous diagnosis of depression related to PSQI (*r*_s_ = 0.295; *p* < 0.05) and to the neurovegetative subscore of BDI (*r*_s_ = 0.293; *p* < 0.05), but not with BDI total score (*r*_s_ = 0.237; *p* = 0.069) having been in psychotherapy related to BDI total score (*r*_s_ = 0.369; *p* < 0.05) and GAD-7 (*r*_s_ = 0.333; *p* < 0.05). Finally, the presence of new symptoms or the worsening of symptoms positively related to FSS (*r*_s_ = 0.311; *p* < 0.05) and to PSQI (*r*_s_ = 0.387; *p* < 0.05).

**Figure 2:**
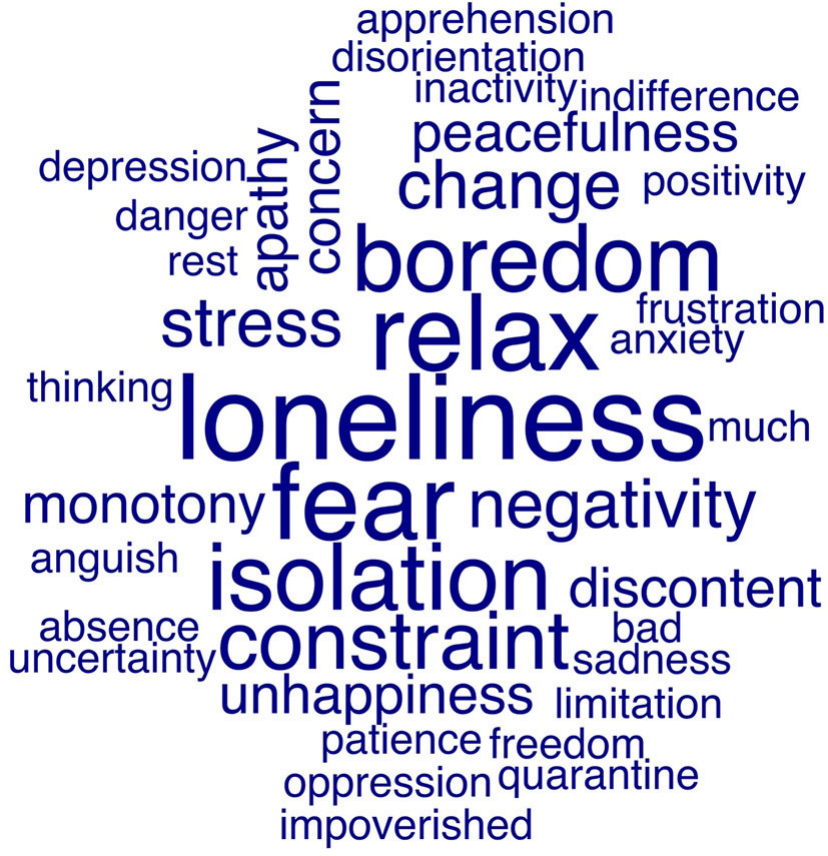
Word-cloud visualization of responses of patients with MS and healthy subjects. We asked subjects to describe their feelings about the impact of COVID-19 pandemic on their life. Then, we performed a word-cloud analysis using the *quanteda* package for RStudio (v 1.1.463). The frequencies of use of a specific word is reflected by the size of the text.

In the HCs group, there was a significant relation between age and PSQI (*r*_s_ = 0.334; *p* < 0.05). A previous diagnosis of depression related to BDI (*r*_s_ = 0.283; *p* < 0.05) and PSQI (*r*_s_ = 0.327; *p* < 0.05). Lastly, there was a significant relation between BDI (*r*_s_ = 0.341; *p* < 0.005), GAD-7 (*r*_s_ = 0.477; *p* < 0.005), and PSQI (*r*_s_ = 0.410; *p* < 0.005) and the self-reported impact of COVID-19 on job. Finally, we performed a word-cloud analysis, whose results are shown in Figure 2.

## Discussion

As the COVID-19 continues to spread across the globe, the uncertainty of contracting a highly contagious disease for which there is no known effective treatment or vaccine, causes insecurity and panic of overexposure among population. This resulted in an “unnatural withdrawal” from society, since, in the past few months, people avoided crowded public places and human interactions. Social distancing, isolation, quarantine, and in some countries, a complete lockdown of social and economic life, produced collaterally a global financial crisis. As easily imaginable, the psychological impact on those hurt by this socioeconomic crisis can be devastating (26–28). During the COVID-19 pandemic in China, 53.8% of the 1,210 respondents in a cross-sectional study rated the psychological impact of the outbreak as moderate or severe, about one-third reported moderate-to-severe anxiety and almost one-fifth reported moderate-to-severe depressive symptoms (29). In a recent review of the impact of quarantine, a high prevalence of negative psychological effects including post-traumatic stress symptoms, confusion, and anger, has emerged (1).

There are particularly concerns about the increased vulnerability of patients living with a chronic disease, and this also includes neurological conditions like MS. It is now generally accepted that MS is a complex multifactorial disease and different environmental factors can influence the functioning of immune system (30). Chronic stress and psychological distress can act as a potential environmental trigger that modulates the disease and its manifestations (13). Just as any other member of the society, PwMS are troubled by the outbreak and are disturbed by the emotional distress and health anxiety caused by the pandemic (31, 32). However, PwMS are known to undertake maladaptive coping strategies in stress conditions (33), and during quarantine, many of them experienced the loss of social support and often were unable to attend cognitive and physical rehabilitation. All this makes patients even more susceptible to the detrimental effects of stress related to the COVID-19 pandemic.

In this study, we focused on the adverse impact of the COVID-19 pandemic on an Italian cohort of PwMS to better understand and measure the psychosocial effects of the viral outbreak in terms of disease course, anxiety, depression, neurovegetative disturbances, and stress in comparison with HCs.

Psychiatric symptoms in MS are highly prevalent (6) and depression have been reported in up to 40% of patients (34), which is above the rates of general population. Not surprisingly, in our survey, PwMS had higher scores of the total BDI and in line with this data, they have increased antidepressive drugs intake. One possible reason is that the pandemic progression and its consequences could cause psychological pressure that hypothetically is added to the level of depression, anxiety, and stress already perceived by patients with MS (31). Indeed, this special population is particularly prone to chronic stress. Significant associations between hypothalamic-pituitary-adrenal (HPA) axis hyperactivity (a common physiological response to stress) and depressive symptoms have been observed, with increased cortisol levels in patients with MS with depressive symptoms compared with nondepressed patients with MS (35). HPA axis activation, influencing glucocorticoid receptors, is important in regulating the serotoninergic system that modulates anxiety and depression (36). Abnormalities in the HPA axis in MS may also contribute to disease progression (35). In our study, one out of five patients reported a worsening of MS disease severity, in particular, sensory disturbances and fatigue that were positively related to FSS and to PSQI. Depression results in increased fatigue and disability scores, disease exacerbations, decreased treatment adherence, and a more aggressive MS course (37, 38). Moreover, several studies have demonstrated that depression in patients with MS determines poor sleep quality and cognitive impairment (31), which can have a negative impact on professional skills and working productivity.

The relationship between job, fatigue, and mood disorder is controverted and has been investigated in different studies (39–43). The abrupt reduction of working hours, together with withdrawn from social life, could have a negative impact on mental and physical health. In fact, we found that there was a significant relationship between unemployment and BDI total score, GAD-7, and PSQI in MS group.

Fatigue is an extremely common symptom in MS and has a great detrimental effect on patients' quality of life. Fatigue in MS seems to have a central rather than peripheral origin, since alterations at neuromuscular level cannot fully explain the extent and severity of the symptom (12, 44). Chauduri (45) defined “central fatigue” as “the failure to initiate and/or sustain attentional tasks (‘mental fatigue') and physical activities (‘physical fatigue') requiring self-motivation,” thus emphasizing the importance of motivation and internal cues over motor or cognitive failures in fatigued patients.

Not surprisingly in our study, two-thirds of patients presented clinically relevant fatigue as measured by FSS, and fatigue was the second most common symptom reported during the lockdown. Cognitive-behavioral therapy (CBT) is a psychological intervention that has demonstrated to have a beneficial effect on fatigue in patients affected by chronic diseases. Of note, one of CBT techniques is activity scheduling, thus emphasizing the importance of daily structured routine in patients dealing with fatigue. In fact, Wicks et al. (46) showed that in PwMS depression and anxiety are at the same time predictors and factors correlated to job instability that in turn influences fatigue perception. The feeling of self-efficacy from a professional point of view is then a critical protective factor against fatigue and mood disorder (47, 48). As said before, the modification of daily routine and working habit during the pandemic could have had a negative impact on patient's health status and specifically on fatigue level.

PwMS often have a wrong cognition about their disease and often believe that fatigue is a symptom totally out of their control and that is better not to engage in potentially tiring activities. Little by little, a fear of becoming always more fatigued occurs and accordingly patients modify their sleep habit increasing rest time, but this in turn has a negative impact on fatigue status (49). Taken together, the complex interplay among the alteration of sleep habit, emotional distress, and the potential psychological vulnerability of patients with MS can contribute to the perception of fatigue and can influence circadian rhythm and autonomic nervous system hyperactivity (50). Indeed, our findings showed that patients had more sleep problems and neurovegetative alterations measured by the PSQI and neurovegetative subscore of BDI, respectively. This consistent pattern of sleep disruption, as seen in different illnesses, points to possible shared underlying framework in which reciprocal interactions of immune, oxidative, and inflammatory stress may drive sleep disturbances across neuroprogressive disorders (51).

Our study also shows a high prevalence of GAD in the general population during the COVID-19 outbreak, which has recently become a major concern. This result is similar to those of a previous study in China during the outbreak (52). Interestingly, the general population reported higher prevalence of GAD symptoms, although not statistically significant, than patients with MS.

Generalized anxiety is characterized by excessive, persistent, and unrealistic worry about everyday things. This worry is pervasive and could be multifocal such as work, finance, family, health, and the future. In fact, in our study, HCs showed higher GAD-7 scores that were mainly determined by the impact that the pandemic had on their job and its foreseeable consequences in economic, social, and family condition. In other words, the COVID-19 outbreak is just one of the factors contributing to generalized anxiety among general population. Instead, viral pandemic is reported to be the main cause of anxiety in patients with MS (32) that are primarily concerned about their health. This data may be related to the patients' “hypochondriac concern” (worry about being infected) (53); patients with MS can feel themselves as particularly vulnerable to infection because, to maintain disease control, in most cases, they require chronic immunotherapy with immunosuppressive or immunomodulatory drugs. At this stage, there is no evidence that being immunosuppressed increases a person's risk of being infected with COVID-19 or developing complications, but there is a theoretical risk of both, and a higher level of health anxiety in this group of patients is expected (31). Accordingly, patients experience increased levels of situational anxiety (i.e., personal health) rather than generalized anxiety.

This study has some limitations that we have to address. First, the differences in the BDI-II and PSQI scores between the two groups, although statistically significant, present a high statistical variability. In this regard, a bigger sample size could have allowed us to consolidate results. Additionally, we did not have any previous neuropsychological evaluation to perform a longitudinal analysis of our cohort. To overcome this limitation, we compared results from PwMS with the results of a cohabitant or partner or family member, so presuming a similar psychological distress deriving from lockdown on both groups. Finally, for the sake of time, we decided not to include in the survey other questionnaires to investigate aspects as quality of life (e.g., the Multiple Sclerosis Quality of Life-54). On the other hand, this study has also some strengths. For instance, the use of an online survey allowed us to have a so-called ecologic momentary assessment (EMA), a well-suited tool to capture patient-reported outcomes (54). EMA makes it possible the sampling of subjects' current behaviors and feelings in real time, in subjects' natural environments (55). Besides, as already said, we were able to make a comparison between two homogeneous groups, thus allowing a better understanding of how differently patients and HCs have dealt with the psychological distress. Lastly, to our knowledge, this is one of the first studies to investigate the psychological status of PwMS during the COVID-19 pandemic. A small cross-sectional study on Iranian patients with MS showed high levels of anxiety, but there was no control group nor a previous neuropsychological evaluation as a reference (32). By contrast, in another study recently published on 67 Italian PwMS in which a previous neuropsychological evaluation was available, anxiety and depression levels did not increase after the pandemic (56). However, also in this case there was no control group and the study population was from one of the least affected region in Italy, something that could explain why anxiety and depression levels did not change pre/postpandemic. Indeed, they found positive effects in terms of quality of life, in particular, concerning sexual satisfaction.

## Conclusions

In our study, we aimed to assess the mental health burden of an Italian cohort of PwMS in comparison with HCs during the COVID-19 outbreak and to explore the potential influencing factors. We identified that the SARS-CoV-2 epidemic had a significant impact on psychological status of PwMS. Indeed, compared with the general population, PwMS presented a higher burden of depressive symptoms, a worse sleep quality and perceived an increase in fatigue level, one of the most disabling symptoms of MS. The COVID-19 epidemic poses a challenge to psychological resilience and the after-effects of the pandemic can be very different between patients affected by chronic diseases and the general population. Research data are needed to better understand neuropsychiatric effects related to the viral pandemic and to develop evidence-driven strategies to improve the mental health of vulnerable groups—patients affected by chronic diseases, children, and adolescents—during the disease outbreaks.

## Data Availability Statement

The raw data supporting the conclusions of this article will be made available by the authors, without undue reservation.

## Ethics Statement

The studies involving human participants were reviewed and approved by Comitato Etico Università Campus Bio-Medico di Roma. The patients/participants provided their written informed consent to participate in this study.

## Author Contributions

FM, MR, MV, GA, and DS wrote the first draft. FM, VD, and FC reviewed and criticized the draft for final submission. All authors contributed to the article and approved the submitted version.

## Conflict of Interest

The authors declare that the research was conducted in the absence of any commercial or financial relationships that could be construed as a potential conflict of interest.
