# Supplementary material for: The Psychological Impact of COVID-19 Pandemic on People With Multiple Sclerosis
Source: Front Neurol. 2020 Oct 30;11:580507. doi: 10.3389/fneur.2020.580507 (PMC7662111; doi:10.3389/fneur.2020.580507)
Supplement: Supplementary file 1 [file Table_1.DOCX]

Checklist for Reporting Results of Internet E-Surveys (CHERRIES)

| ITEM CATEGORY | EXPLANATION |
| --- | --- |
| **Design** | The study involved a convenience sample. Patients affected by Multiple Sclerosis (MS) and healthy controls aged over 18 years were included in this study. |
| **IRB** | Approval. The study has been approved by the Università Campus Bio-Medico di Roma IRB.  Informed consent. Participants read the Participant Disclosure Form, but it did not require a signature due to the anonymous nature of the survey. Participants received written instructions on how to complete the survey, length of time required to complete the survey and data storage information.  Data protection. No personally identifying information was collected. |
| **Development and pre-testing** | The survey was developed through reviewing the literature. A pilot-testing was performed on a small cohort of subjects. |
| **Recruitment process** | Survey type. The data was collected using a closed survey.  Contact mode. Subjects were initially contacted through phone calls. Then an email containing the link to the survey was sent to participants.  Advertising the survey. The link to the survey was embedded in the email invitation to participate. Only subjects received the link to the survey. |
| **Survey administration** | Web/E-mail. This study utilized an Internet-based survey research design  Context. We conducted this survey using Google Form, Google LLC (Mountain View, CA, USA).  Mandatory/voluntary. The survey was voluntary.  Incentives. None. Time/Date. Enrollment started on April 27^th^, 2020. Last subject was enrolled on May 25^th^, 2020 Randomization of items or questionnaire. N/A  Adaptive questioning. N/A  Number of items. There were 107 items in total.  Number of screens. Twelve screens.  Completeness check. Manual completeness checks were done during the data analysis phase.  Review step. Participants were able to review and change their answers. A back button and a review step were included before subjects could send the results. |
| **Response rates** | Unique site visitor. Patients could use the link embedded in the email invitation just once.  View rate. N/A  Participation rate. Participation rate was calculated as the ratio between number of people who received the link to the survey divided by the ones who agreed to participate  Completion rate. Completion rate was not calculated. |
| **Preventing multiple entries** | Cookies used. Cookies were not used. IP check. IP addresses were not collected. Log file analysis. The study did not include a log file analysis.  Registration. N/A |
| **Analysis** | Handling of incomplete surveys. Also incomplete surveys were analyzed.  Questionnaires submitted with an atypical timestamp. N/A  Statistical correction. N/A |
